# Warmer Temperature Accelerates the Aging-Dependent Decrease in Female Ovary Size, Delays Male Accessory Gland Development, and Accelerates Aging-Dependent Changes in Reproductive Gene Expression in *Anopheles gambiae* Mosquitoes

**DOI:** 10.3390/insects16090921

**Published:** 2025-09-02

**Authors:** Lindsay E. Martin, Tania Y. Estévez-Lao, Megan I. Grant, Norbu Y. Shastri, Julián F. Hillyer

**Affiliations:** Department of Biological Sciences, Vanderbilt University, Nashville, TN 37232, USA; lindsay.e.martin@vanderbilt.edu (L.E.M.); tania.y.estevez-lao@vanderbilt.edu (T.Y.E.-L.); megan.i.grant@vanderbilt.edu (M.I.G.); norbu.y.shastri@vanderbilt.edu (N.Y.S.)

**Keywords:** reproduction, mosquito, male, female, temperature, aging, senescence, warming, ovaries, male accessory glands

## Abstract

In most mosquito species, reproduction requires mating between the female and the male, followed by the female blood-feeding, completing oogenesis, and laying eggs. Warmer environmental temperature and aging both reduce mosquito fecundity and fertility, and warmer temperature accelerates the aging-dependent decline in reproduction such that reproductive impairment manifests earlier in life. To shed light on how this warming-based acceleration of reproductive senescence occurs, we investigated how temperature (27 °C, 30 °C, and 32 °C) and aging interactively shape female and male reproductive tissue size in the African malaria mosquito, *Anopheles gambiae*. In blood-fed females, we discovered that warmer temperature accelerates the aging-dependent decrease in the size of the ovaries but not the spermatheca. In males, we discovered that warmer temperature lessens and delays the aging-dependent increase in the size of the male accessory glands but not the testes. Next, we measured the expression of reproductive genes in females and males. In female reproductive tissues, warmer temperature accelerates the aging-dependent decrease in the expression of vitellogenin and the aging-dependent increase in the expression of *MISO* and *HPX15*. In male reproductive tissues, warmer temperature accelerates an aging-dependent decrease in the expression of *Plugin*, *TGase3*, *phLP*, and *CYP315A1*. Altogether, these data shed light on how physical and transcriptional changes underpin the warming-based acceleration of an aging-dependent decline in mosquito fecundity and fertility.

## Simple Summary

A mosquito population can only be maintained when their members reproduce. When the environmental temperature is warmer, mosquito reproduction is less efficient. Likewise, reproductive efficiency is lower when mosquitoes are older. Importantly, at warmer temperatures, the aging-dependent decline in reproduction is accelerated. To shed light on how warmer temperature accelerates the pace of reproductive deterioration, we investigated how temperature (27 °C, 30 °C and 32 °C) modifies the aging-related changes in reproductive tissue size and gene expression in the African malaria mosquito, *Anopheles gambiae*. We discovered that warmer temperature accelerates the aging-dependent decrease in the size of the ovaries of female mosquitoes, while delaying and lessening the early development of the male accessory glands of male mosquitoes. Moreover, warmer temperature accelerates aging-dependent changes in the expression of reproductive genes in both female and male mosquitoes. In summary, warmer temperature physically and transcriptionally alters reproductive tissues in ways that accelerate the aging-dependent decline in reproductive efficiency, resulting in fewer eggs laid by each female. These changes may affect how mosquito populations are maintained and how these mosquitoes transmit disease-causing pathogens.

## 1. Introduction

To complete oogenesis and reproduce, most female mosquitoes must complete two events: (i) mate with a male to receive the sperm needed for fertilization, and (ii) take a blood meal to obtain the nutrients needed to produce eggs. In preparation for oogenesis, female mosquitoes ready themselves by developing their ovaries through several stages: (i) previtellogenesis, which occurs within the first 72 h after eclosion; (ii) reproductive arrest, which occurs around 72 h after eclosion and before the first blood meal; and (iii) vitellogenesis, which begins after mating and blood feeding and entails the synthesis of yolk protein precursors (YPPs) in the fat body and their transport to growing oocytes [1,2,3,4,5].

The sesquiterpenoid juvenile hormone (JH) primarily regulates previtellogenesis, while the ecdysteroid 20-hydroxyecdysone (20E) primarily regulates vitellogenesis [1,5,6,7,8,9,10,11,12,13,14,15,16]. Specifically, stimulated by metamorphosis and by nutritional sensing, the corpora allata produces JH until 48–50 h post eclosion, which triggers the maturation of primary ovarian follicles and primes the fat body for YPP synthesis [7,10,11,12,13,14,15,16,17,18,19]. During mating (and usually prior to blood feeding), males transfer a mating plug into the female’s atrium that contains sperm and a large amount of 20E [20]. This sexually transferred 20E regulates oogenesis and oviposition and induces transcriptional changes that result in refractoriness to mating [20,21,22,23,24,25,26].

When a female ingests blood, JH titers decrease, and the ovaries synthesize 20E from blood-meal-derived cholesterol [5]. The female- and male-derived 20E induces expression of YPPs in the fat body, which are secreted into the hemolymph and transported to developing ovarian follicles [1,2,5,27]. Moreover, the male-derived 20E regulates the expression of Mating-Induced Stimulator of Oogenesis (*MISO*), which stimulates the accumulation of lipids in developing oocytes [22]. As follicles develop, the oocytes become distinguishable from nurse cells, and the yolk progressively increases in size as proteins and lipids accumulate until the oocyte has reached its full length, resulting in an oblong egg [4,7,28,29,30].

The ability to reproduce is affected by extrinsic factors such as environmental temperature, and intrinsic factors such as the age of the mosquito [31]. Because mosquitoes are ectothermic poikilotherms and their body temperature conforms to the temperature of the environment, warmer environmental temperature increases metabolism, alters body composition and size, and weakens immunity [32,33,34,35,36,37,38,39,40,41,42]. Warmer temperature also decreases egg hatching, fecundity, and fertility [31,37,41,43,44,45]. How warmer environmental temperature affects the reproductive ability of male mosquitoes is unknown, but in fruit flies and flour beetles, warmer temperature reduces sperm abundance, competitiveness, viability, and fertilization success [46,47,48].

Aging also affects reproductive success because, like in most organisms, the physiological condition of mosquitoes progressively deteriorates with time, a phenomenon known as senescence [49,50,51,52]. Aging causes a decline in body condition, immunity, and reproductive capacity [31,34,35,36,38,53,54,55,56,57,58]. Pertaining to reproduction, aging in female mosquitoes reduces blood feeding propensity, survival after a blood meal, oviposition success, fecundity, and fertility [31,56,57,58]. Because female mosquitoes must survive long enough to mate, take a blood meal, complete oogenesis and lay eggs, and because older females are less likely to survive and reproduce, the age at which a female mates and blood feeds impacts reproductive success. In males, aging beyond 10 days also reduces the production of sperm, the synthesis of seminal proteins, and the ability to inseminate [59,60,61,62,63]. Therefore, the age at which mating occurs also dictates reproductive success.

Warmer temperature quickens senescence in mosquitoes [31,34,35,36,38,55]. For example, warmer temperature accelerates an aging-dependent weakening in immunity and body condition and shortens lifespan [34,35,36,38,55]. Importantly, warmer temperature delays oviposition and accelerates an aging-dependent decline in female fecundity, fertility, and survival, with females effectively becoming infertile at 32 °C, regardless of age [31]. However, how warmer temperature accelerates reproductive senescence remains unknown for either sex. Mosquito population size is a critical determinant of disease transmission risk [64]. Therefore, identifying how warmer temperature accelerates reproductive senescence may identify targets that can be manipulated to reduce mosquito populations.

Here, we investigated how warmer temperature and aging interact to modify reproductive tissues in the African malaria mosquito, *Anopheles gambiae*. We discovered that warmer temperature accelerates the aging-dependent decrease in ovary size in blood-fed females and delays the early development of male accessory glands (MAGs). Moreover, warmer temperature accelerates aging-dependent changes in the expression of reproductive genes in both female and male reproductive tissues.

## 2. Materials and Methods

### 2.1. Mosquito Rearing, Feeding, and Dissection

A colony of *A. gambiae* (Giles *sensu stricto*, G3 strain; Diptera: Culicidae) was maintained in an environmental chamber at standard rearing conditions: 27 °C, 75% humidity, and a 12 h:12 h light–dark photoperiod [31]. The eggs from this colony were hatched in environmental chambers held at either 27 °C, 30 °C, or 32 °C with the same humidity and photoperiod. These temperatures simulate warming environmental temperatures that mosquitoes may experience in nature [65,66]. Upon hatching, larvae were fed a 2.8:1 mixture of koi food and baker’s yeast daily. Each day, pupae were separated, and eclosed adults were fed 10% sucrose daily. Female and male adults of the same age were caged together until experimentation.

**Figure 1:**
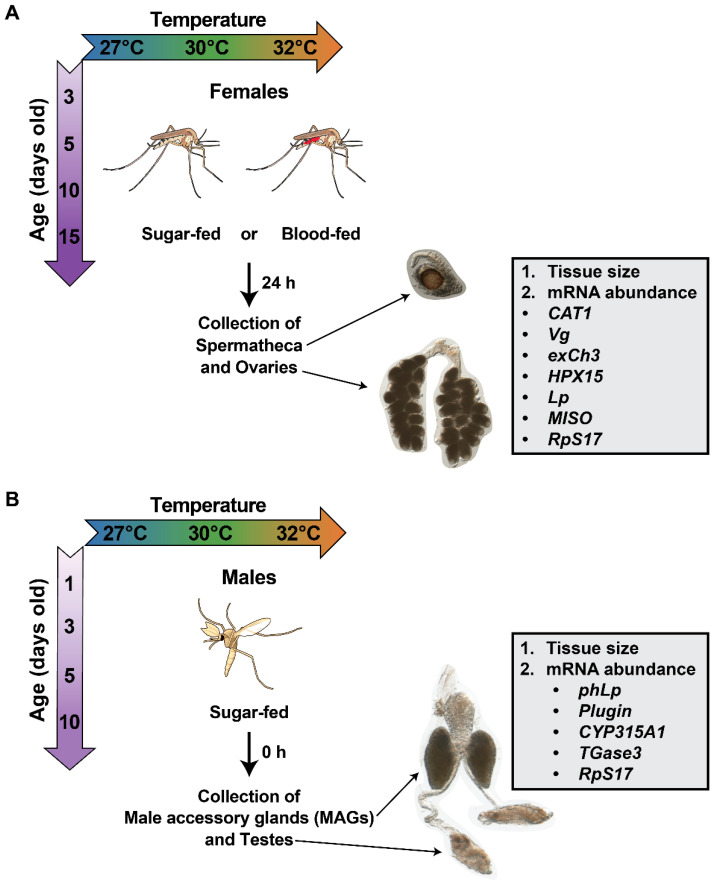
Experimental overview to determine the effects of warmer temperature, aging, and their interaction on female and male reproductive tissues. (**A**) Females were reared at 27, 30, or 32 °C and received a sugar or blood meal at 3, 5, 10, or 15 days of age. At 24 h after feeding, female spermathecae and ovaries were collected and assayed for tissue size or gene expression. (**B**) Males were reared at 27, 30, or 32 °C and fed sugar. At 1, 3, 5, or 10 days of age, male accessory glands (MAGs) and testes were dissected and assayed for tissue size or gene expression. Images of reproductive tissues are not to scale.

Experiments were conducted at each temperature on blood-fed females, and on sugar-fed females and males (Figure 1A,B). Experiments on females were initiated at 3, 5, 10, or 15 days of age whereas experiments on males were initiated at 1, 3, 5, or 10 days of age (Figure 1B). These ages were selected because females begin seeking a blood meal at approximately 3 days of age [5,14,16], and males complete sexual maturation and mate after 1 day post emergence [67,68,69,70,71].

To blood feed females, mosquitoes were starved for 1 h and then offered a blood meal for 30 min. Defibrinated sheep blood (Hemostat Laboratories, Dixon, CA, USA) was loaded into a Hemotek membrane feeder (Hemotek Ltd., Blackburn, UK) with a Parafilm membrane (Amcor, Neenah, WI, USA) and heated to 37 °C. Blood feeding was stimulated by breathing onto the mosquitoes two to three times throughout the 30 min period.

Female mosquitoes were anesthetized on ice and dissected in sterile PBS at 24 h after blood or sugar feeding, and males were dissected at the age when the experiment was started. The 24 h time point for females was selected to allow for vitellogenesis to begin in blood-fed females, capturing differences in ovary size prior to egg laying [5]. To dissect females, the spermathecae and ovaries were removed by grasping the 8th abdominal segment with forceps and gently pulling. To dissect males, the MAGs and testes were removed by grasping the claspers with forceps and gently pulling. Forceps and 0.20 mm diameter minutien insect pins were used to separate and clean the tissues. The female and male tissues were then immediately used to measure size or to extract RNA.

### 2.2. Measuring Reproductive Tissue Size

Upon dissection, reproductive tissues were immersed in a drop of PBS on a glass microscope slide and imaged on a Nikon SMZ1500 stereomicroscope connected to a Nikon Digital Sight DS-Fi1 CCD Color Camera and Nikon Advanced Research NIS-Elements software v4.13.00 (Nikon, Tokyo, Japan). A micrometer slide was also imaged with every batch of photographs for linear length calibration (Meiji Techno America, Campbell, CA, USA).

Using ImageJ v1.5.4d (U.S. National Institutes of Health, Bethesda, MD, USA), we first calibrated the scale using the “set scale” tool on the micrometer images. Then, we measured the length and width of each ovary, or the diameter of each spermathecae. Similarly, the length and width of each MAG and testis were measured. Each measurement was made in triplicate, and the average of the three measurements was calculated for each individual tissue. Then, for each mosquito, the average length and width of the pair of tissues (ovaries, MAGs, and testes; there is only one spermatheca in each female) was calculated to generate a single value for each feature per mosquito (e.g., ovary width). If a mosquito only had one measurable tissue from the pair, this tissue was used to determine the size.

For females, approximately 25 spermathecae and 20 pairs of ovaries were measured per temperature-age-feeding group combination, which were collected across 3–5 biological trials. In total, 603 spermathecae and 489 pairs of ovaries were measured. For males, approximately 28 pairs of MAGs and 7 pairs of testes were measured per temperature-age combination, which were collected across 3 biological trials. The delicate nature of the testes, which are fragile and often broke apart during dissection in a way that prevented measurement, limited the sample size of the testes. In total, 333 pairs of MAGs and 85 pairs of testes were measured.

Each tissue measurement was analyzed independently. For ovary length, ovary width, and spermathecae diameter, the effects of temperature, age, feeding group (sugar or blood) and their two- and three-way interactions, were analyzed using generalized linear regressions (gaussian family with log link). For MAG length and width, the effects of temperature, age, and their two-way interaction were analyzed using generalized linear regressions (gaussian family with log link). For testes length and width, the effects of temperature and age were compared using descriptive statistics because of the smaller sample size. Feeding group was not a variable for males because they only ingest sugar. Models were fit and assessed using the “glmmTMB” and “DHARMA” packages in R [72,73,74]. For each measurement, model selection was followed by a type-II ANOVA (Wald chi-square tests) with Kenward-Roger approximation of degrees of freedom and by Sidak-adjusted post hoc comparisons of estimated marginal means using the “emmeans” package [75,76]. Averages, variances, datapoints, Chi-square values, degrees of freedom, and *p* values are presented in the figures. Raw data, model information, additional post hoc comparisons, and R code are presented in the supplemental data files (Supplementary Files S1–S3).

### 2.3. Measuring Reproductive Tissue mRNA Abundance by Quantitative Real-Time PCR

At each temperature, females that took a blood meal at 5 or 15 days of age were dissected at 24 h after blood feeding, and males that ingested sugar were dissected at 3, 5, or 10 days of age. For females, reproductive tissues from ~14 mosquitoes—spermathecae, ovaries, and the tissues that connect them—were pooled in 200 µL of TRIzol, homogenized, and RNA was isolated as per manufacturer’s protocol (Invitrogen, Carlsbad, CA, USA). For males, reproductive tissues of ~20 mosquitoes—MAGs, testes, and the tissues that connect them—were similarly pooled and RNA isolated. RNA was further purified using the RNeasy Mini Kit (Qiagen, Valencia, CA, USA), and up to 5 µg of RNA was used for cDNA synthesis using the SuperScript III First-Strand Synthesis System for RT-PCR and Oligo (dT)_20_ primers (Applied Biosystems, Foster City, CA, USA). Using the cDNA as template, gene specific primers (Table S1), and the Power SYBR Green PCR Master Mix (Applied Biosystems, Foster City, CA, USA), RT-qPCR was performed on a Bio Rad CFX Connect Real-Time Detection System (Hercules, CA, USA). Relative mRNA abundance was determined via the 2^−ΔΔCT^ method [77], using the ribosomal protein housekeeping gene *RpS7* as the reference, and *RpS17* as a control. Fold-change in mRNA abundance was calculated using the coolest temperature and the youngest age as the baseline (27 °C–5-day-old for females and 27 °C–3-day-old for males). For each temperature-age combination and sex, three independent biological trials were conducted, and each trial had two or three technical replicates. In total, we measured mRNA abundance in 18 female samples (derived from ~252 females) and 27 male samples (derived from ~540 males).

To determine whether mRNA abundance differed across temperature-age groups, a non-parametric Kruskal–Wallis Chi-square rank sum test was conducted for each gene, followed by Dunn’s post hoc comparisons. The results from the Kruskal–Wallis test are presented in the figures, while post hoc comparisons are presented in the supplement (Supplementary Data File S2).

To visualize which genes share similar expression patterns across temperature-age groups, average fold change values were log-transformed for each gene. Using the R package “pheatmap” (v1.0.12) [78], log-transformed fold change values were plotted on a heatmap, in which Euclidian distances were used to cluster genes (heatmap rows) by expression pattern similarity. Heatmap colors show similarity and deviation from the mean gene expression across temperature-age groups.

## 3. Results

### 3.1. Neither Warmer Temperature, Aging, nor Feeding Affect the Size of a Female’s Spermatheca

We first assessed how warmer temperature and aging affect the size of the female’s sperm storage organ, called the spermatheca [79]. While some insects have more than one spermatheca, *A. gambiae* females only have one [79,80].

**Figure 2:**
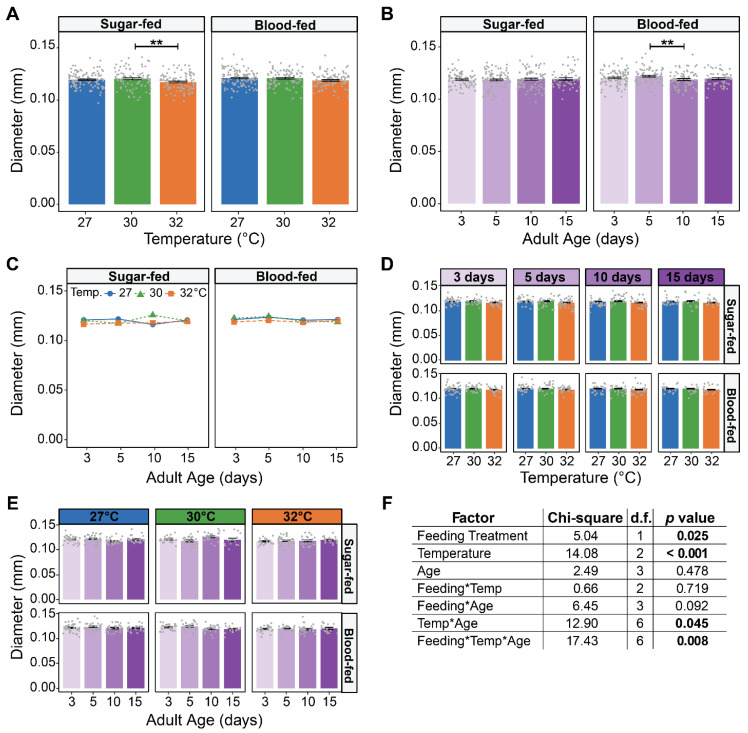
Warmer temperature and aging do not affect spermatheca size. (**A**) Mean spermatheca diameter of females reared at each temperature that took a sugar or blood meal, irrespective of age. (**B**) Mean spermatheca diameter of females that took a sugar or blood meal at each age, irrespective of temperature. (**C**) Interaction plot showing the mean spermatheca diameter of females that took a sugar or blood meal at each temperature within each age group. (**D**,**E**) Mean spermatheca diameter of females that took a sugar or blood meal at each temperature within each age group (**D**) or at each age within each temperature group (**E**). (**F**) Statistical outcomes determined by a generalized linear regression model (gaussian family with log link) followed by a type-II ANOVA Wald Chi-square test with Kenward–Roger approximation of degrees of freedom. The same data are plotted multiple ways: main effects of temperature and age (within feeding group) are shown in (**A**) and (**B**), respectively, and unaggregated data are shown in (**D**,**E**). In (**A**,**B**,**D**,**E**), bars represent means, whiskers indicate the SEM, and grey dots show individual female data points. Sidak-adjusted post hoc comparisons of means in (**A**,**B**) are indicated by asterisks: ** *p* < 0.01.

Relative to sugar-fed females, the spermatheca of blood-fed females was 1.13% larger, indicating that blood feeding does not meaningfully affect its size. Relative to mosquitoes at 27 °C, the spermatheca of both sugar-fed and blood-fed mosquitoes at 32 °C was ~1.8% smaller, indicating that rearing temperature does not affect its size (Figure 2A). The size of the spermatheca was also similar at all ages tested (Figure 2B). However, there was a slight interaction between temperature, age, and feeding treatment (Figure 2C–F). This interaction is driven by sugar-fed mosquitoes reared at 30 °C having a spermatheca that was slightly larger at 10 days old, but the difference is small. In summary, spermatheca size is not meaningfully affected by blood feeding, warmer temperature, aging, or their interaction.

### 3.2. Warmer Temperature Accelerates the Aging-Dependent Decrease in the Size of the Ovaries of Blood-Fed Females

**Figure 3:**
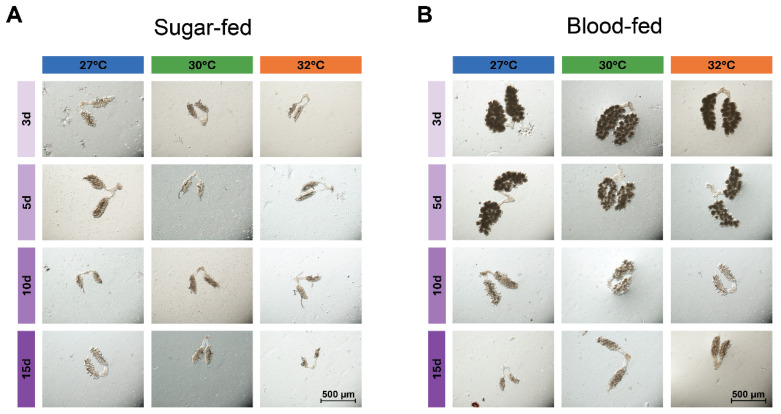
Effects of warmer temperature, aging, and their interaction on female ovaries. (**A**,**B**) Representative images of ovaries from females that were reared at 27, 30, or 32 °C and were sugar-fed (**A**) or blood-fed (**B**) at 3, 5, 10, or 15 days of age. Ovaries were dissected 24 h after feeding. Quantitative analyses of ovary size are presented in Figure 4 and Figure S1.

**Figure 4:**
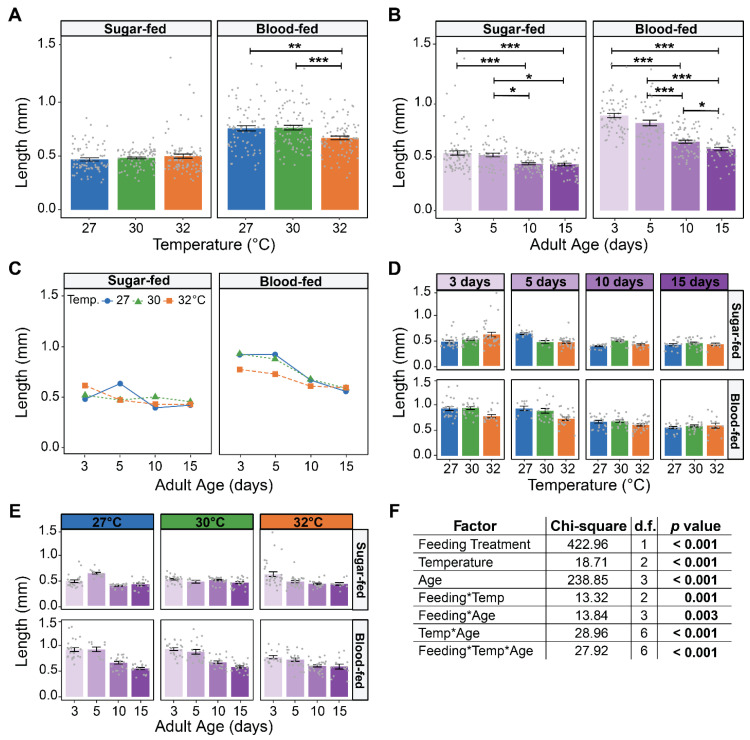
Warmer temperature accelerates the aging-dependent decrease in ovary length in blood-fed females. (**A**) Mean ovary length of females reared at each temperature that took a sugar or blood meal, irrespective of age. (**B**) Mean ovary length of females that took a sugar or blood meal at each age, irrespective of temperature. (**C**) Interaction plot showing the mean ovary length of females that took a sugar or blood meal at each temperature within each age group. (**D**,**E**) Mean ovary length of females that took a sugar or blood meal at each temperature within each age group (**D**) or at each age within each temperature group (**E**). (**F**) Statistical outcomes determined by a generalized linear regression model (gaussian family with log link) followed by a type-II ANOVA Wald Chi-square test with Kenward–Roger approximation of degrees of freedom. The same data are plotted multiple ways: main effects of temperature and age (within feeding group) are shown in (**A**) and (**B**), respectively, and unaggregated data are shown in (**D**,**E**). In (**A**,**B**,**D**,**E**), bars represent means, whiskers indicate the SEM, and grey dots show individual female data points. Sidak-adjusted post hoc comparisons of means in (**A**,**B**) are indicated by asterisks: *** *p* < 0.001, ** *p* < 0.01, and * *p* < 0.05.

Relative to sugar feeding, blood feeding increased ovary size (Figure 3, Figure 4 and Figure S1). Across all temperatures and ages, blood-fed females had ovaries that were 51% longer and 90% wider than sugar-fed females (Figure 3, Figure 4 and Figure S1). Additionally, females that blood-fed at 3 and 5 days of age had ovaries that were similar in size, color and translucency at all temperatures, but the ovaries of older mosquitoes were smaller and more translucent, with fewer discernible follicles (Figure 3).

Temperature did not affect ovary size in sugar-fed mosquitoes, but the warmest temperature of 32 °C reduced ovary size in blood-fed mosquitoes (Figure 4A and Figure S1A). Specifically, ovary size in blood-fed females was similar between 27 °C and 30 °C, but 12% shorter and 21% narrower at 32 °C.

Aging reduced ovary size in both sugar-fed and blood-fed females, irrespective of temperature (Figure 4B and Figure S1B). Sugar-fed females that were 15 days of age had ovaries that were 20% shorter and 28% narrower than those that were 3 days of age. Similarly, 15-day-old mosquitoes that blood-fed had ovaries that were 35% shorter and 54% narrower than those that received a blood meal at 3 days of age.

Warmer temperature and aging interacted to shape ovary size in both sugar-fed and blood-fed females, and this interactive effect depended on whether they ingested blood or not (Figure 4C–F and Figure S1C–F). In sugar-fed females, at 27 °C ovary size increased as the mosquito aged from 3 to 5 days old and then decreased, whereas at 32 °C, there was a progressive decrease with age. However, in blood-fed females, at 27 °C, ovary size was similar between 3 and 5 days old followed by a steep aging-related decrease, whereas at 32 °C, there was a constant but more gradual aging-related decrease.

In summary, ovaries are smallest at 32 °C in blood-fed females, and aging decreases the size of the ovaries regardless of diet. Moreover, warmer temperature accelerates the aging-dependent decrease in the size of the ovaries of blood-fed females.

### 3.3. Warmer Temperature Lessens and Delays the Increase in the Size of Male Accessory Glands That Occurs Early in Life

**Figure 5:**
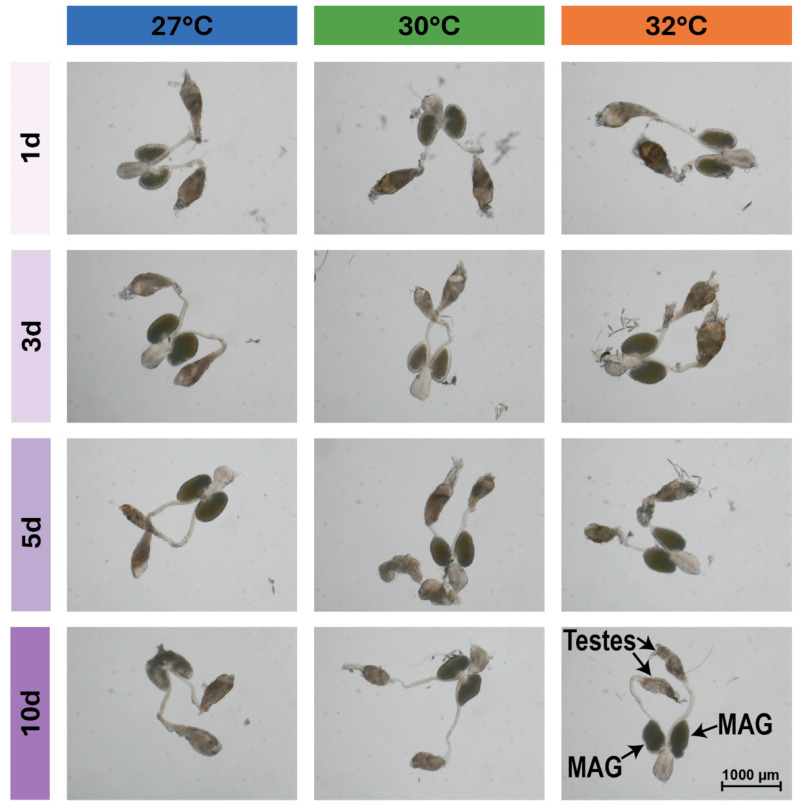
Effects of warmer temperature, aging, and their interaction on male accessory glands and testes. Representative images of male accessory glands (MAGs) and testes from males that were reared at 27, 30, or 32 °C and dissected at 1, 3, 5, or 10 days of age. Quantitative analyses of MAGs are presented in Figure 6 and Figure S2 and of testes in Figure 7 and Figure S3.

**Figure 6:**
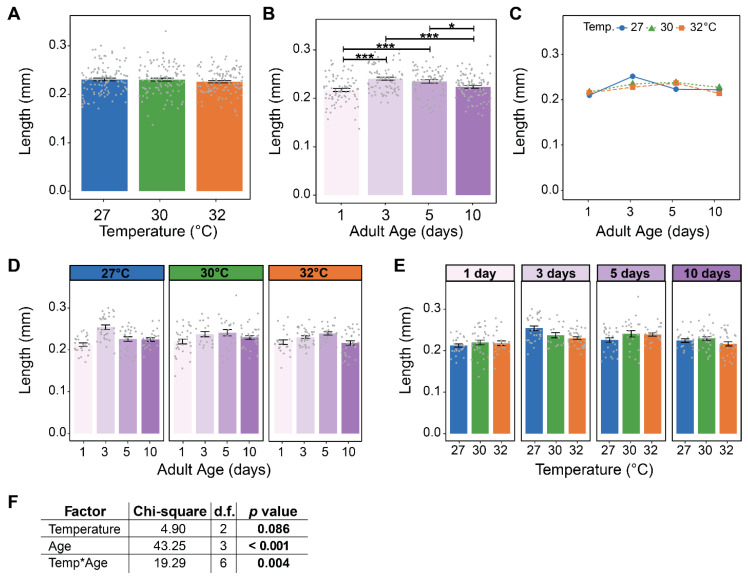
Warmer temperature delays and lessens the initial increase in male accessory gland (MAG) length. (**A**) Mean MAG length of males reared at each temperature, irrespective of age. (**B**) Mean MAG length of males at each age, irrespective of temperature. (**C**) Interaction plot showing the mean MAG length of males at each temperature within each age group. (**D**,**E**) Mean MAG length of males at each temperature within each age group (**D**) or at each age within each temperature group (**E**). (**F**) Statistical outcomes determined by a generalized linear regression model (gaussian family with log link) followed by a type-II ANOVA Wald Chi-square test with Kenward–Roger approximation of degrees of freedom. The same data are plotted multiple ways: main effects of temperature and age (within feeding group) are shown in (**A**) and (**B**), respectively, and unaggregated data are shown in (**D**,**E**). In (**A**,**B**,**D**,**E**), bars represent means, whiskers indicate the SEM, and grey dots show individual male data points. Sidak-adjusted post hoc comparisons of means in (**A**,**B**) are indicated by asterisks: *** *p* < 0.001 and * *p* < 0.05.

Warmer temperature did not affect MAG size, but aging non-linearly shaped MAG size (Figure 5, Figure 6A,B and Figure S2). Specifically, MAG length increased by 11% from 1 to 3 days of age, plateaued between 3 and 5 days of age, and decreased by 5% from 5 to 10 days of age (Figure 5 and Figure 6B). MAG width was affected in a similar manner, but the increase between 1 and 3 days of age was less pronounced (Figure 5 and Figure S2B).

Although warmer temperature alone did not shape MAG size, warmer temperature and aging interacted to alter MAG size (Figure 6C–F and Figure S2C–F). At 27 °C, MAG size increased between 1 and 3 days of age and then decreased, whereas at 30 °C and 32 °C the increase was smaller and slower, peaking at 5 days of age before decreasing. Moreover, although temperature alone did not affect MAG size, at 27 °C, the MAGs were largest in 3-day-old mosquitoes, but at 30 °C and 32 °C, they were larger in 5-day-old mosquitoes.

In summary, the size of MAGs increases from 1 to 3 days of age, plateaus from 3 to 5 days of age, and then decreases. Moreover, warmer temperature lessens and delays the increase in MAG size that occurs early in life.

### 3.4. Warmer Temperature Increases the Size of the Testes

**Figure 7:**
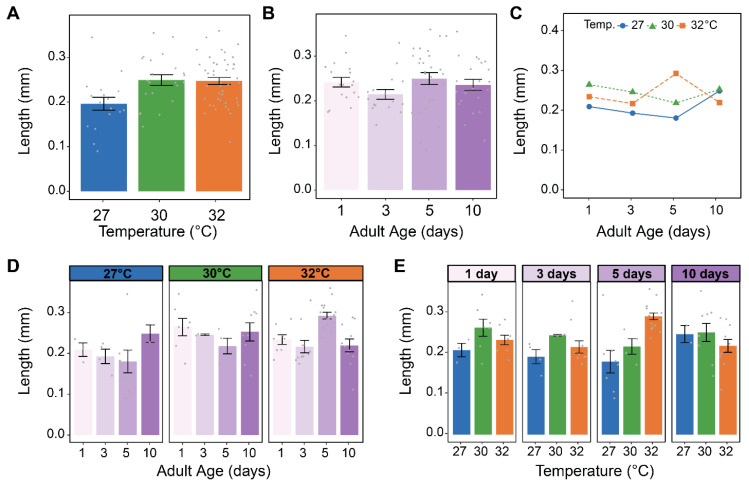
Warmer temperature increases testes length. (**A**) Mean testes length of males reared at each temperature, irrespective of age. (**B**) Mean testes length of males at each age, irrespective of temperature. (**C**) Interaction plot showing the mean testes length at each temperature within each age group. (**D**,**E**) Mean testes length at each temperature within each age group (**D**) or at each age within each temperature group (**E**). The same data are plotted multiple ways: main effects of temperature and age (within feeding group) are shown in (**A**) and (**B**), respectively, and unaggregated data are shown in (**D**,**E**). In (**A**,**B**,**D**,**E**), bars represent means, whiskers indicate the SEM, and grey dots show individual male data points.

Warmer temperature increased the size of the testes, which was mostly manifested in increased length (Figure 5, Figure 7A and Figure S3A). Testes were 27% longer at 30 °C than at 27 °C, but there was no meaningful size difference between 30 °C and 32 °C. Aging non-uniformly affected the size of the testes (Figure 7B and Figure S3B). Specifically, testes length was smallest at 3 days of age but was relatively similar at all other ages, whereas testes width was the same from 1 to 5 days of age but smallest at 10 days of age. Finally, warmer temperature and aging do not appear to interact to shape testes length or width (Figure 7C–E and Figure S3C–E).

### 3.5. In Female Reproductive Tissues, Warmer Temperature Accelerates an Aging-Dependent Decrease in Vg Expression but an Increase in MISO and HPX15 Expression

To determine whether warmer temperature and aging individually and interactively shape the expression of genes involved in reproduction within the female reproductive tissues (spermathecae, ovaries, and their connecting tissue), we assessed 5-day-old and 15-day-old adult females at each temperature at 24 h after blood feeding. These ages were selected because female fertility is greatest at 5 days of age and lowest at 15 days of age [31]. We assayed genes involved in several aspects of female reproduction: (i) Na^+^/K^+^/Ca^2+^ exchanger 3 (*exCh3*) and heme peroxidase 15 (*HPX15*), both of which protect sperm [80,81]; (ii) vitellogenin (*Vg*) and lipophorin (*Lp*), which are genes that encode YPPs [2,3,82,83]; (iii) mating-induced stimulator of oogenesis (*MISO*) that regulates mating and lipid distribution [22,26,84,85,86,87,88]; and (iv) catalase 1 (*CAT1*) that mediates oxidative stress [89,90,91].

**Figure 8:**
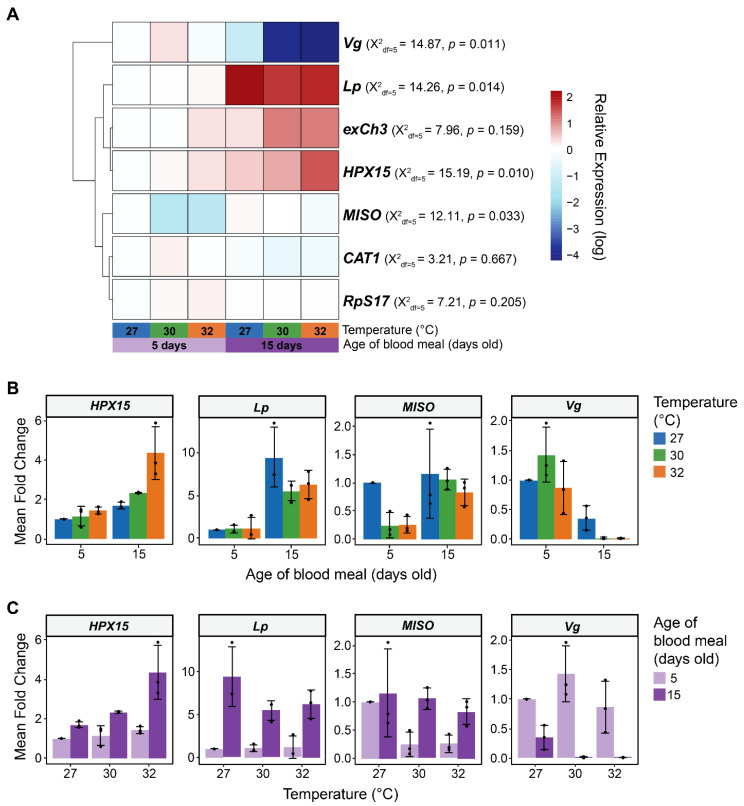
Effects of warmer temperature, aging, and their interaction on gene expression in female reproductive tissues. (**A**) Heatmap illustrates the relative expression of each gene across temperature-age groups. Average fold change values, relative to females reared at 27 °C receiving a blood meal at 5 days old, were log-transformed for each gene. Gene rows were clustered by expression pattern similarity. For each gene, statistical differences among temperature-age groups were detected using a non-parametric Kruskal–Wallis Chi-square rank sum test. (**B**,**C**) Mean fold change in female reproductive genes that differed across temperature-age groups. Column heights mark the mean mRNA fold change, relative to females reared at 27 °C receiving a blood meal at 5 days old, at each temperature within each age group (**B**) or at each age within each temperature group (**C**). Whiskers indicate the SEM, and black dots show the values for each trial. *RpS7* was used as the reference gene, and *RpS17* was used as the control gene.

The expression of female reproductive genes was strongly shaped by aging (Figure 8). Specifically, aging from 5 to 15 days, irrespective of temperature, increased mRNA abundance of *HPX15*, *MISO*, and *Lp* by 134%, 102%, and 546%, respectively. Opposingly, aging from 5 to 15 days decreased mRNA abundance of *Vg* by 88%.

Warmer temperature also shaped the expression of female reproductive genes (Figure 8). Specifically, *HPX15* mRNA abundance was 114% higher at 32 °C than at 27 °C, *MISO* was 50% lower at 32 °C than at 27 °C, and *Lp* and *Vg* did not meaningfully change with temperature. The mRNA abundance of *exCh3* and *CAT1* did not differ across temperature-age groups, and as expected, the mRNA abundance of the ribosomal gene, *RpS17*, also did not change (Figure 8A and Figure S4).

Warmer temperature and aging interactively altered the expression of *Vg*, *HPX15* and *MISO*, but not *Lp*, *CAT1*, or *exCh3* (Figure 8B,C). Specifically, in 5-day-old females, mRNA abundance of *Vg* resembled an inverted parabola as the temperature changed from 27 °C to 32 °C, whereas in 15-day-olds, it was a precipitous drop. Additionally, the warming-based increase in *HPX15* mRNA abundance was much more pronounced in 15-day-old mosquitoes than in 5-day-old mosquitoes. Finally, the aging-dependent increase in *MISO* occurred at 30 °C and 32 °C, but not at 27 °C. In summary, warmer temperature accelerates the aging-dependent changes in mRNA abundance in female reproductive tissues.

### 3.6. In Male Reproductive Tissues, Warmer Temperature Accelerates the Aging-Dependent Decrease in the Expression of Plugin, TGase3, phLP, and CYP315A1

We next conducted a similar analysis on the reproductive tissues of males (MAGs, testes and their connecting tissues) in 3-, 5-, and 10-day-old adults. These ages were selected because males have completed sexual maturation by 3 days of age but have a shorter lifespan than females [67,68,69,70,71,92], and so, this range captures males that are sexually mature and mating. We measured the transcription of genes involved in several aspects of male reproduction: (i) Plugin (*Plugin*) and transglutaminase-3 (*TGase3*) that form the mating plug [21,93,94]; (ii) lysophospholipase (*phLp*) involved in lipid metabolism [95,96]; and (iii) a 2-hydroxylase enzyme (*CYP315A1*) involved in 20E biosynthesis [20,21,97].

**Figure 9:**
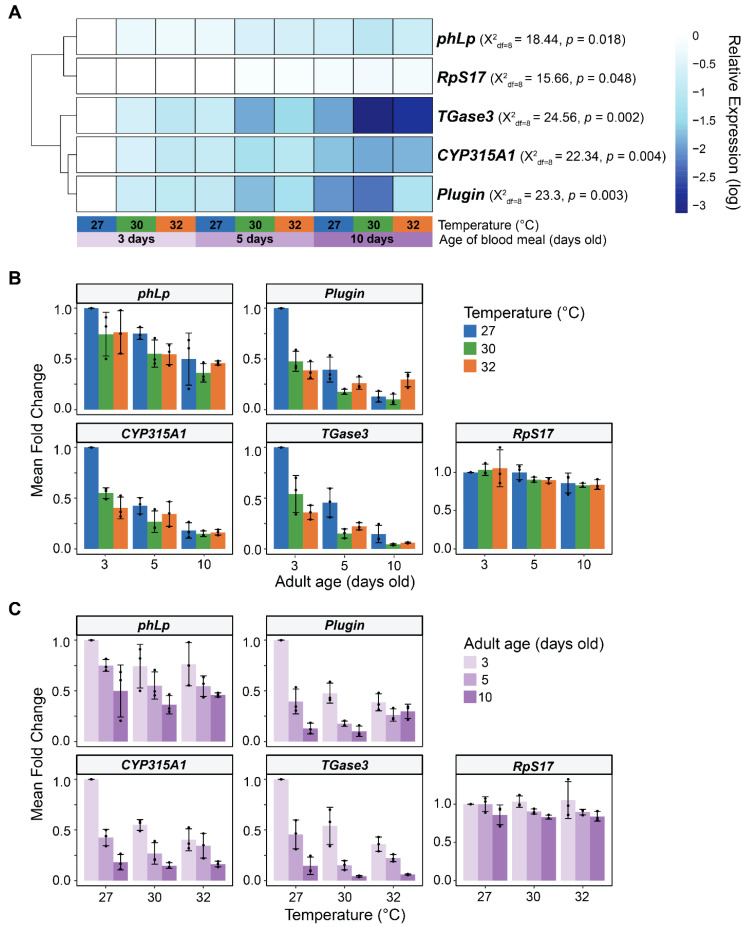
Effects of warmer temperature, aging, and their interaction on gene expression in male reproductive tissues. (**A**) Heatmap illustrates the relative expression of each gene across temperature-age groups. Average fold change values, relative to males reared at 27 °C dissected at 3 days old, were log-transformed for each gene. Gene rows were clustered by expression pattern similarity. For each gene, statistical differences among temperature-age groups were detected using a non-parametric Kruskal–Wallis Chi-square rank sum test. (**B**,**C**) Mean fold change in male reproductive genes that differed across temperature-age groups. Column heights mark the mean mRNA fold change, relative to males reared at 27 °C dissected at 3 days old, at each temperature within each age group (**B**) or at each age within each temperature group (**C**). Whiskers indicate the SEM, and black dots show values for each trial. *RpS7* was used as the reference gene, and *RpS17* was used as the control gene.

Like in females, the expression of male reproductive genes was strongly shaped by aging (Figure 9). Specifically, aging from 3 to 10 days decreased mRNA abundance of *phLp*, *Plugin*, *CYP315A1*, and *TGase3* by 47%, 72%, 75%, and 87%, respectively. The mRNA abundance of the control ribosomal gene, *RpS17*, decreased slightly (18%) with aging.

Warmer temperature also decreased the expression of all four genes (Figure 9). Specifically, the mRNA abundance of *phLp*, *Plugin*, *CYP315A1*, and *TGase3* was 21%, 38%, 43%, and 60% lower at 32 °C than at 27 °C, respectively.

Warmer temperature accelerated the aging-dependent decrease in the expression of *Plugin*, *CYP315A1*, and *TGase3* (Figure 9B,C). For example, at both 27 °C and 32 °C, *TGase3* mRNA abundance decreased by 85% and 83%, respectively, as males aged from 3 to 10 days. However, in 3-day-old mosquitoes, *TGase3* mRNA abundance was already 64% lower at 32 °C than at 27 °C, indicating that the decline in *TGase3* expression begins earlier in life when the temperature is warmer.

In summary, expression of male reproductive genes decreases with aging and warmer temperature. Moreover, warmer temperature accelerates the aging-dependent decline in expression.

## 4. Discussion

**Figure 10:**
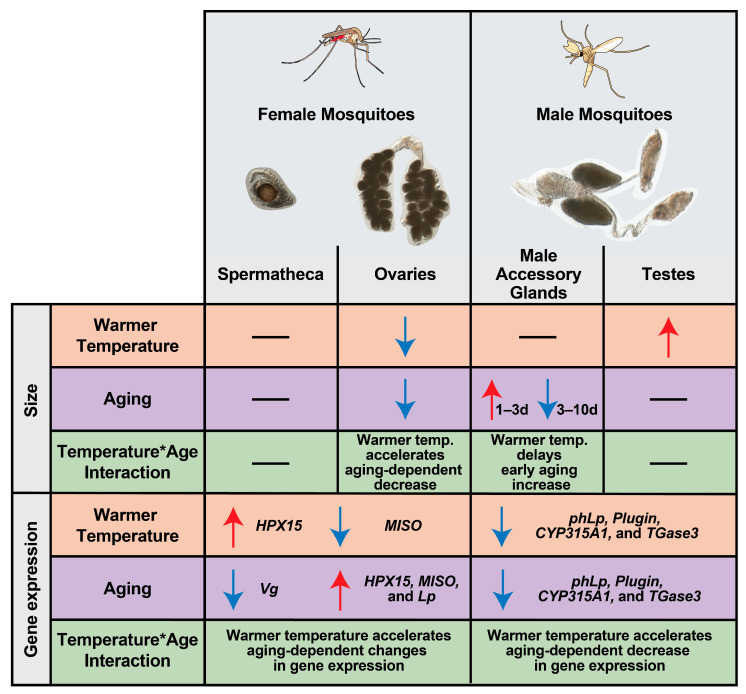
Graphical summary illustrating how warmer temperature accelerates the aging-dependent changes in female and male reproductive tissues.

Warmer temperature accelerates reproductive senescence in female mosquitoes, reducing fecundity and fertility earlier in life [31]. Here, we demonstrate that warmer temperature modifies the aging-dependent changes in the size of female and male reproductive tissues and the expression of reproductive genes in ways that reflect declining fecundity and fertility (Figure 10).

Warmer temperature accelerates the aging-based decrease in oviposition success, fecundity and fertility [31]. Here, we discovered that warmer temperature accelerates an aging-dependent decrease in the size of ovaries of blood-fed females, providing a physical explanation for the previously observed reduction in reproductive ability. Ovaries may be smaller because they contain fewer follicles, have delayed follicle development during vitellogenesis, have degenerated follicles, or a combination of these factors, any of which would reduce egg production. Similar to our findings, in other dipterans, warmer temperature reduces the size of ovaries, and warmer temperature increases the death of early germline cysts and of follicles undergoing vitellogenesis [98,99,100]. Mosquitoes reared at 27 °C and 32 °C are only marginally different in body size [35], so the warming-based reduction in the size of the ovaries occurs independently of body size. Moreover, aging also decreases the size of ovaries in sugar-fed females. Given that previtellogenesis is completed by 3 days of age [5,14,16], this suggests that the aging-based deterioration of the ovaries begins prior to blood feeding. As dipterans age, reactive oxygen species cause oxidative damage that reduces fecundity [89,90,101,102]. This aging-based increase in oxidative damage is exacerbated by warmer temperature when females take a blood meal [103,104]. Moreover, mosquitoes at warmer temperatures have lower protein content and fewer teneral reserves, which are also depleted with aging [35,105,106]. So, although the body composition of sugar-fed mosquitoes reared at 27 °C and 32 °C is relatively similar [35], females at the warmer temperature may divert more blood-meal-acquired proteins and lipids toward replenishing their energy stores [1,19,105,106], having a deleterious impact on ovary development. Thus, when the temperature is warmer, ovaries are smaller and the aging-based deterioration occurs faster, resulting in an accelerated deterioration of the egg production machinery [31].

Previously, we observed that warmer temperature accelerates an aging-dependent decline in fertility, ultimately reducing the number of hatched larvae [31]. We uncovered that warmer temperature accelerates an aging-dependent decrease in the expression of *Vg* in female reproductive tissues. Moreover, we also observed that decreased *Vg* expression correlates with increased *Lp* expression, suggesting that excess lipids are transported into the reproductive tissues when the temperature is warmer. Reduced *Vg* expression and increased *Lp* expression can cause accelerated embryonic arrest and infertility by (i) impairing amino acid incorporation into the developing oocytes, (ii) preventing oocyte melanization, and (iii) causing excess lipids to accumulate in the ovaries [107].

The warming based acceleration of an aging-dependent decrease in fecundity and fertility may also be driven by a reduction in 20E, which positively regulates *Vg*, *Lp*, *MISO*, and *HPX15* [1,5,9,22,80,108]. We previously observed that warmer temperature reduces blood meal size [31], and smaller blood meals provide less cholesterol, which could reduce 20E levels given that cholesterol is the building block of 20E. Alternatively, warmer temperature may reduce the amount of 20E transferred from the male to the female during copulation, which reduces expression of 20E-responsive genes in the female and decreases their ability to lay eggs [23]. This hypothesis is supported by our observation that warmer temperature accelerates an aging-dependent decrease in the expression of the male enzyme *CYP315A1* involved in 20E synthesis. Moreover, we uncovered that warmer temperature delays the increase in the size of the MAGs that produce 20E [20,26], potentially reducing the production of sperm and MAG proteins [60,109,110]. Therefore, we hypothesize that the warming-based acceleration of an aging-dependent decline in fecundity and fertility may be due to both a female- and male-20E-mediated decline in *Vg* expression.

The Target of Rapamycin (TOR) signaling pathway detects the levels of amino acids in the hemolymph and also positively regulates *Vg* expression [1,9,111]. Warmer temperature and aging both deplete the teneral reserves of proteins, lipids, and carbohydrates [35,37,41,112,113,114,115,116], and females that have low teneral reserves use their first blood meals to replenish somatic energy stores prior to diverting energy to egg production [106,117,118,119,120]. Thus, at warmer temperatures, depletion of teneral reserves may occur faster, thereby decreasing TOR-mediated *Vg* expression. Fewer amino acids incorporated into developing oocytes led to reduced fertility [107], so this likely contributes to the warming-based acceleration of an aging-dependent decline in fecundity and fertility.

Upon mating, the female nourishes the collected sperm inside the spermatheca for the remainder of her reproductive lifespan [79,121,122]. Here, the warming-based acceleration of the aging-dependent decrease in the expression of mating plug and lipid metabolism genes in males (*Plugin*, *TGase3*, and *phLp*) suggests that males transfer fewer sperm-protecting components during copulation. Moreover, warmer temperature accelerates an aging-dependent increase in the expression of *HPX15* in females, suggesting that the heme peroxidase that protects long-term fertility is more essential when the temperature is warmer [80].

YPPs, such as vitellogenin (*Vg*) and lipophorin (*Lp*), were previously thought to only be produced in the fat body. More recent studies have demonstrated that YPPs are also expressed in the reproductive tissues of some insects, including mosquitoes [6,83,87,123,124,125,126]. Here, we detected expression of *Vg* and *Lp* in female reproductive tissues. However, the reproductive tissue samples collected from both females and males may contain a small amount of adhered fat body, which would contribute to the *Vg* and *Lp* mRNA detected [127]. Regardless, it is unknown how warmer temperature and aging, individually or interactively, alter the expression of these genes in their primary site of production—the fat body—and this should be the focus of future experimentation.

Although we uncovered that warmer temperature accelerates an aging-dependent decrease in the size of ovaries, the size of the spermatheca remained unchanged. The spermatheca is made of chitin and resilin that together form a thick cuticle-based structure that stores and protects sperm upon mating. Because the spermatheca is fully developed and sclerotized prior to adult eclosion [79,121], and because body size is relatively similar when mosquitoes are reared 27 °C versus 32 °C [35], it stands to reason that spermatheca size should not change with blood feeding, warmer temperature, or aging.

Beyond warmer temperature and aging, other factors may affect the development of reproductive tissues. This study focused on *Anopheles gambiae*, but reproduction may differ across mosquito species and strains [62,128]. For example, *Aedes aegypti* males transfer JH to females during mating, whereas *A. gambiae* males transfer 20E to females during mating [20,62,128,129,130]. Moreover, other abiotic and biotic factors may also interact to affect reproductive ability. For example, mosquitoes must protect themselves against desiccation in low-humidity environments [131], and low humidity decreases egg production and hatching [132,133,134,135].

In summary, in blood-fed females, warmer temperature accelerates an aging-dependent decrease in the size of the ovaries and modifies the changes in reproductive gene expression that occur with aging. In males, warmer temperature lessens and delays the increase in the size of the male accessory glands and accelerates an aging-dependent decrease in reproductive gene expression. Given that warmer temperature accelerates reproductive senescence [31], these data shed light on how physical and transcriptional changes underpin the warming-based acceleration of an aging-dependent decline in mosquito fecundity and fertility.

## Data Availability

All data and analyses supporting this manuscript are available in the main text or provided as Supplementary Materials.

## Supplementary Materials

The following supporting information can be downloaded at: https://www.mdpi.com/article/10.3390/insects16090921/s1, Figure S1. Warmer temperature accelerates the aging-dependent decrease in ovary width in blood-fed females; Figure S2. Warmer temperature delays the initial increase in male accessory gland (MAG) width; Figure S3. Warmer temperature increases testes width; Figure S4. Effects of warmer temperature, aging, and their interaction on the expression of additional genes in female reproductive tissues; Table S1. Gene names, IDs, and primers used; File S1. Raw Data; File S2. Processed Data; File S3. R Code.

## Abbreviations

The following abbreviations are used in this manuscript:

MAGs	Male Accessory Glands
Temp	Temperature
d.f.	Degrees of freedom
20E	20-hydroxyecdysone
JH	Juvenile hormone
