# Supplementary material for: Warmer Temperature Accelerates the Aging-Dependent Decrease in Female Ovary Size, Delays Male Accessory Gland Development, and Accelerates Aging-Dependent Changes in Reproductive Gene Expression in Anopheles gambiae Mosquitoes
Source: Insects. 2025 Sep 2;16(9):921. doi: 10.3390/insects16090921 (PMC12470671; doi:10.3390/insects16090921)
Supplement: Supplementary file 1 [file insects-16-00921-s001.zip › Martin et al Insects 2025 Supp Figs and Table R1.pdf]

insects

MDPI

Article – Supplementary Figures and Table

Warmer temperature accelerates the aging-dependent decrease in female ovary size, delays male accessory gland development, and accelerates aging-dependent changes in reproductive gene expression in *Anopheles gambiae* mosquitoes

Lindsay E. Martin [1], Tania Y. Estévez-Lao [1], Megan I. Grant [1], Norbu Y. Shastri [1] and Julián F. Hillyer [1*]

[1] Department of Biological Sciences, Vanderbilt University, Nashville, Tennessee, United States of America
* Correspondence: julian.hillyer@vanderbilt.edu

Supplementary Figures and Table:

Figure S1. Warmer temperature accelerates the aging-dependent decrease in ovary width in blood-fed females.

Figure S2. Warmer temperature delays the initial increase in male accessory gland (MAG) width.

Figure S3. Warmer temperature increases testes width.

Figure S4. Effects of warmer temperature, aging, and their interaction on the expression of additional genes in female reproductive tissues.

Table S1. Gene names, IDs, and primers used.

 http://doi.org/10.3390/insects16090921

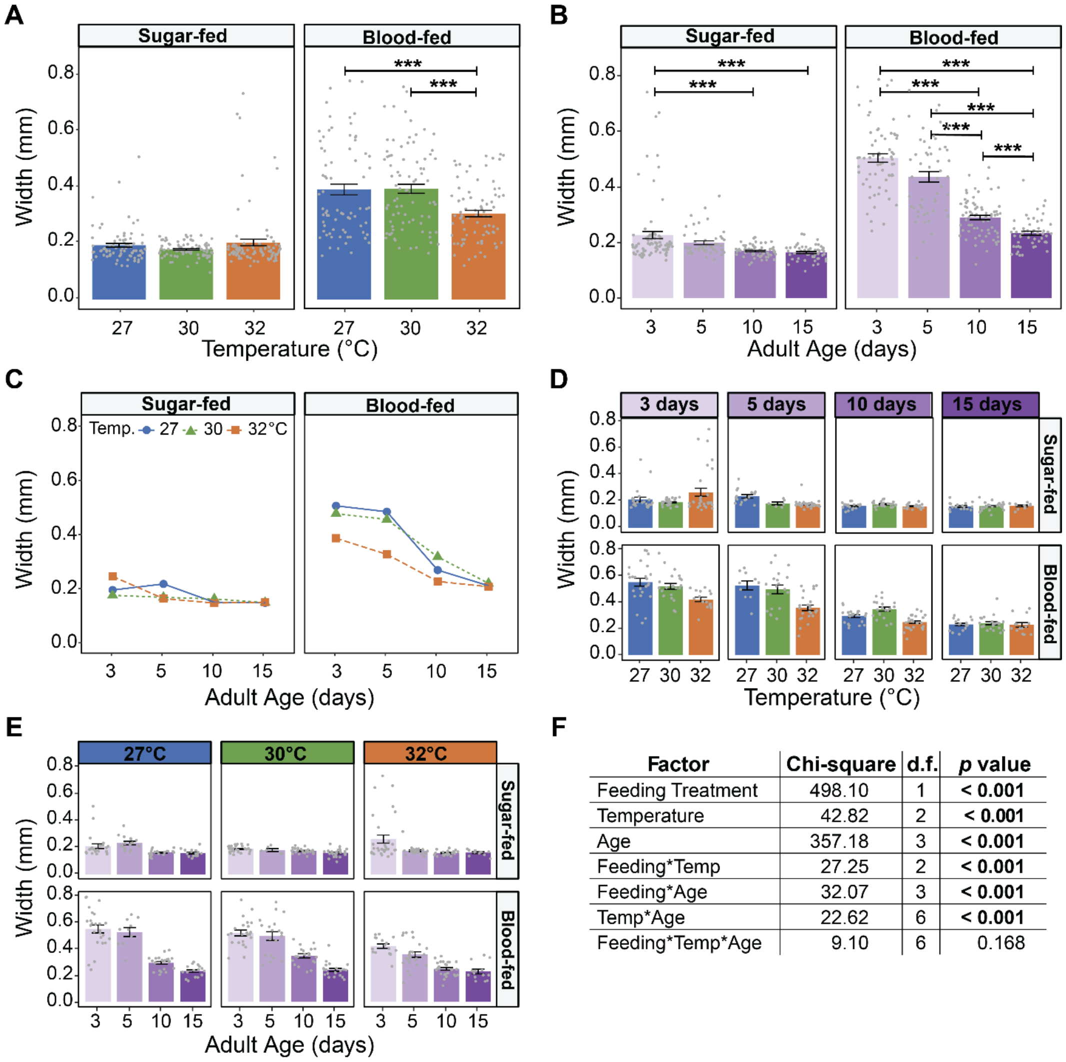

Factor	Chi-square	d.f.	*p* value
Feeding Treatment	498.10	1	**< 0.001**
Temperature	42.82	2	**< 0.001**
Age	357.18	3	**< 0.001**
Feeding*Temp	27.25	2	**< 0.001**
Feeding*Age	32.07	3	**< 0.001**
Temp*Age	22.62	6	**< 0.001**
Feeding*Temp*Age	9.10	6	0.168

Figure S1. Warmer temperature accelerates the aging-dependent decrease in ovary width in blood-fed females. (**A**) Mean ovary width of females reared at each temperature that took a sugar or blood meal, irrespective of age. (**B**) Mean ovary width of females that took a sugar or blood meal at each age, irrespective of temperature. (**C**) Interaction plot showing the mean ovary width of females that took a sugar or blood meal at each temperature within each age group. (**D-E**) Mean ovary width of females that took a sugar or blood meal at each temperature within each age group (**D**) or at each age within each temperature group (**E**). (**F**) Statistical outcomes determined by a generalized linear regression model (gaussian family with log link) followed by a type-II ANOVA Wald Chi-square test with Kenward–Roger approximation of degrees of freedom. The same data are plotted multiple ways: main effects of temperature and age (within feeding group) are shown in (**A**) and (**B**), respectively, and unaggregated data are shown in (**D-E**). In (**A-B**) and (**D-E**), bars represent means, whiskers indicate the SEM, and circles show individual female data points. Sidak-adjusted post-hoc comparisons of means in (**A**) and (**B**) are indicated by asterisks: *** $p < 0.001$, ** $p < 0.01$, and * $p < 0.05$.

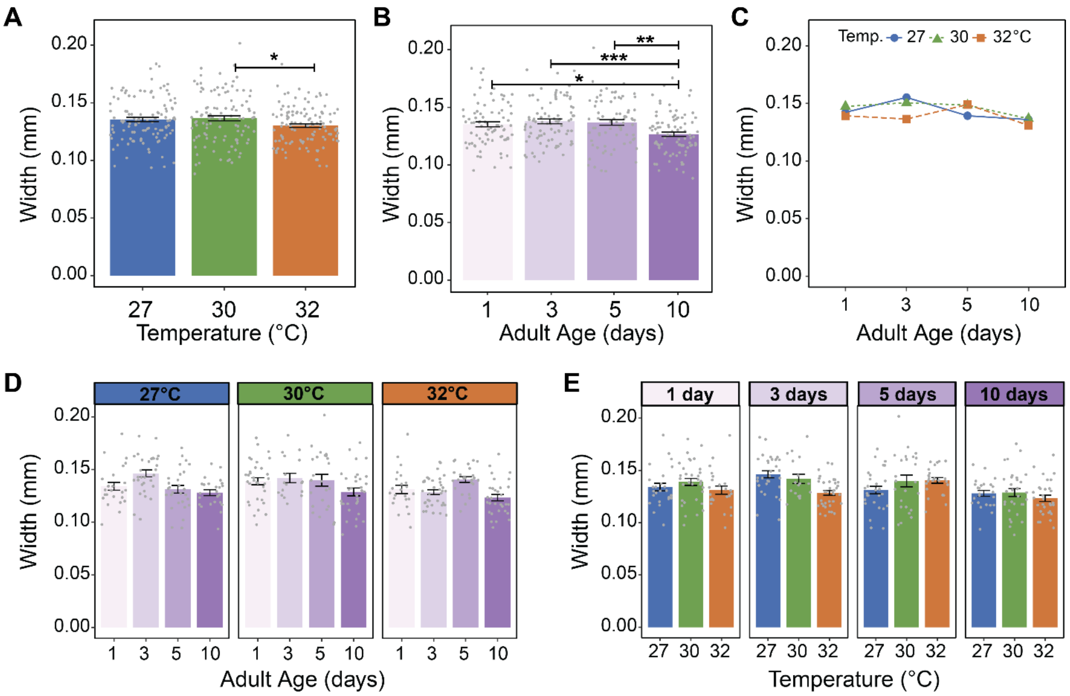

F

Factor	Chi-square	d.f.	*p* value
Temperature	8.58	2	**0.014**
Age	21.68	3	**< 0.001**
Temp*Age	16.45	6	**0.012**

Figure S2. Warmer temperature delays the initial increase in male accessory gland (MAG) width. (**A**) Mean MAG width of males reared at each temperature, irrespective of age. (**B**) Mean MAG width of males at each age, irrespective of temperature. (**C**) Interaction plot showing the mean MAG width of males at each temperature within each age group. (**D-E**) Mean MAG width of males at each temperature within each age group (**D**) or at each age within each temperature group (**E**). (**F**) Statistical outcomes determined by a generalized linear regression model (gaussian family with log link) followed by a type-II ANOVA Wald Chi-square test with Kenward–Roger approximation of degrees of freedom. The same data are plotted multiple ways: main effects of temperature and age (within feeding group) are shown in (**A**) and (**B**), respectively, and unaggregated data are shown in (**D-E**). In (**A-B**) and (**D-E**), bars represent means, whiskers indicate the SEM, and circles show individual male data points. Sidak-adjusted post-hoc comparisons of means in (**A**) and (**B**) are indicated by asterisks: *** $p < 0.001$, ** $p < 0.01$, and * $p < 0.05$.

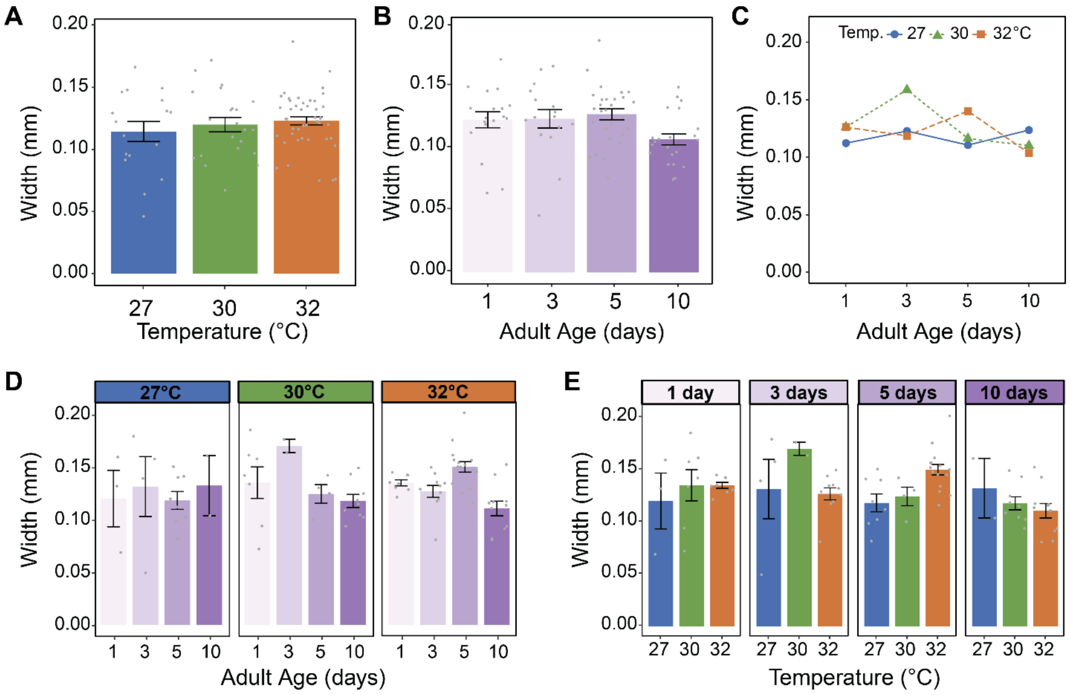

Figure S3. Warmer temperature increases testes width. (**A**) Mean testes width of males reared at each temperature, irrespective of age. (**B**) Mean testes width of males at each age, irrespective of temperature. (**C**) Interaction plot showing the mean testes width at each temperature within each age group. (**D-E**) Mean testes width at each temperature within each age group (**D**) or at each age within each temperature group (**E**). The same data are plotted multiple ways: main effects of temperature and age (within feeding group) are shown in (**A**) and (**B**), respectively, and unaggregated data are shown in (**D-E**). In (**A-B**) and (**D-E**), bars represent means, whiskers indicate the SEM, and circles show individual male data points.

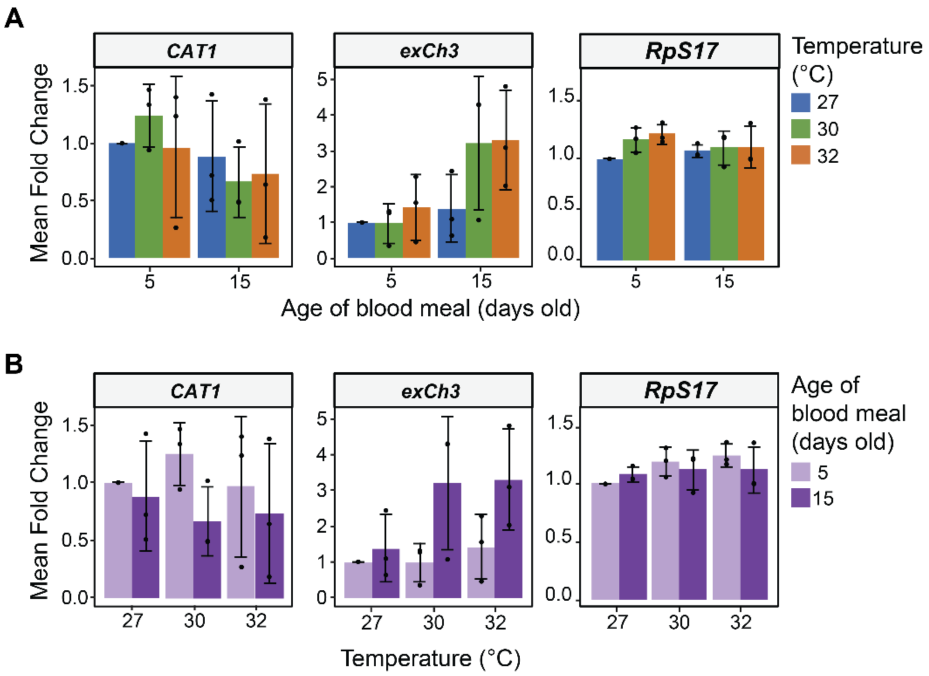

Figure S4. Effects of warmer temperature, aging, and their interaction on the expression of additional genes in female reproductive tissues. (**A-B**) Mean fold change of female reproductive genes that did not change across temperature-age groups. Column heights mark the mean mRNA fold change, relative to females reared at 27°C receiving a blood meal at 5 days old, at each temperature within each age group (**A**) or at each age within each temperature group (**B**). Whiskers indicate the SEM, and circles show values for each trial. *RpS7* was used as the reference gene, and *RpS17* was used as the control gene.

Supplementary Table S1. Gene names, IDs, and primers used.

				Amplicon (bp)	
Sex Assayed	Gene Name	Vectorbase ID*	Nucleotide sequence (5'-3')	Tran-script	Genomic
Females	*Lp*	AGAP001826	F: ACACCAAGGACCAGTCGAAG<br>R:GTTCTGCAGCACGAAGAAGT	182	249
Females	*Vg*	AGAP004203	F:TCCACCCCGACTTTGACTAC<br>R:CGAGTTCATGAAGTGCGAGT	209	283
Females	*MISO*	AGAP002620	F:AGACGATGGAGGGACTGATG<br>R:GGATTCGCTTTCGTGCTG	80	80
Females	*HPX15*	AGAP013327	F:ATGCAACATCCGCGAAACAC<br>R:GATGGGTAAGCATCTCGGGG	153	253
Females	*exCh3*	AGAP010973	F:TGTTCTCACTCGGATTGCCC<br>R:CGCAGCGTATACTTGGCAAC	169	169
Females	*CAT1*	AGAP004904	F:ACGGAAAACAAATCCCATCA<br>R:GTCGATCAGGTGAACGTCCT	188	7533
Males	*CYP315A1*	AGAP00284	F:CCTTTACCGAATGCCAACAG<br>R: AATTCGATGATAGCCGTTGC	202	274
Males	*TGase3*	AGAP009099	F:CTGCACAATCCCACCTACGA<br>R: CGACCAGTAGATCACGTCCG	180	180
Males	*phLp*	AGAP002397	F:TGGGACTGGGAGACGAAGAA<br>R:GTCAGCTTTGTCACATCGCC	215	292^
Males	*Plugin*	AGAP009368	F: TCGTTCCGTTGTATGGTGGT<br>R:AGGGATTTGCGGGATATGCT	215	215
Both	*RpS7*	AGAP010592	F:GACGGATCCCAGCTGATAAA<br>R: GTTCTCTGGGAATTCGAACG	132	281
Both	*RpS17*	AGAP004887	F:GACGAAACCACTGCGTAACA<br>R: TGCTCCAGTGCTGAAACATC	153	264

* Vectorbase IDs were obtained from the AgamP4 assembly in www.vectorbase.org.

^:2/3 of the reverse primer sequence binds at the end of the exon and amplifies
